# A prognostic nomogram for long-term major adverse cardiovascular events in patients with acute coronary syndrome after percutaneous coronary intervention

**DOI:** 10.1186/s12872-021-02051-0

**Published:** 2021-05-22

**Authors:** Shuting Kong, Changxi Chen, Gaoshu Zheng, Hui Yao, Junfeng Li, Hong Ye, Xiaobo Wang, Xiang Qu, Xiaodong Zhou, Yucheng Lu, Hao Zhou

**Affiliations:** 1 Department of Cardiology, The First Affiliated Hospital of Wenzhou Medical University, Wenzhou, 325000 Zhejiang China; 2 Cardiac Interventional Center, The First Affiliated Hospital of Wenzhou Medical University, Wenzhou, 325000 Zhejiang China; 3 Affiliated Jinhua Hospital, Zhejiang University School of Medicine, Jinghua, 321000 Zhejiang China; 4 The First Clinical Medical College of Wenzhou Medical University, Wenzhou Medical University, Wenzhou, 325000 Zhejiang China

**Keywords:** ACS Nomogram predict MACE Risk

## Abstract

**Background:**

Accurate prediction of major adverse cardiovascular events (MACEs) is very important for the management of acute coronary syndrome (ACS) patients. We aimed to construct an effective prognostic nomogram for individualized risk estimates of MACEs for patients with ACS after percutaneous coronary intervention (PCI).

**Methods:**

This was a prospective study of patients with ACS after PCI from January 2013 to July 2019 (n = 2465). After removing patients with incomplete clinical information, a total of 1986 patients were randomly divided into evaluation (n = 1324) and validation (n = 662) groups. Predictors included in the nomogram were determined by a multivariate Cox proportional hazards regression model based on the training set. Receiver operating characteristic (ROC) curves and calibration curves were used to assess the discrimination and predictive accuracy of the nomogram, which were then compared with those of the classic models. The clinical utility of the nomogram was assessed by X-tile analysis and Kaplan–Meier curve analysis.

**Results:**

Independent prognostic factors, including lactate level, age, left anterior descending branch stenosis, right coronary artery stenosis, brain natriuretic peptide level, and left ventricular ejection fraction, were determined and contained in the nomogram. The nomogram achieved good areas under the ROC curve of 0.712–0.762 in the training set and 0.724–0.818 in the validation set and well-fitted calibration curves. In addition, participants could be divided into two risk groups (low and high) according to this model.

**Conclusions:**

A simple-to-use nomogram incorporating lactate level effectively predicted 6-month, 1-year, and 4-year MACE incidence among patients with ACS after PCI.

## Background

According to the statistical results of the World Health Organization, coronary artery diseases such as acute coronary syndrome (ACS) have become some of the most frequent causes of death worldwide [1]. ACS includes acute ST-segment elevation myocardial infarction (STEMI), non-ST-segment elevation myocardial infarction (NSTEMI), and unstable angina pectoris (UA) [2]. Percutaneous coronary intervention (PCI) remains the most effective treatment for ACS [3, 4]. However, the incidence of MACE in patients with different risk factors varies, especially in high-risk cases [5, 6]. A predictive model would allow physicians to better identify patients at elevated risk, which would facilitate a more personalized approach to managing these cases.

Several cardiovascular disease risk and prognosis assessment tools have been established in different populations to guide clinical practice [7–14]. GRACE and Thrombolysis in Myocardial Infarction (TIMI) risk scores are recommended in the guidelines for predicting cardiovascular outcomes (short- and medium-term) for patients with ACS [7, 15–17]. The CADILLAC risk score is used to predict 30-day and 1-year mortality after PCI for acute myocardial infarction (AMI) [8]. Unfortunately, clinical risk stratification for long-term MACEs for patients with ACS after PCI is not well defined.

In recent years, an increasing number of studies have focused on laboratory testing indicators to predict the prognosis of diseases. Several recent studies have shown that lactate level is an independent prognostic factor that is useful for identifying patients at high risk [18–21]. Our purpose in designing a nomogram incorporating lactate was to provide a tool for the clinical evaluation of patients with ACS undergoing PCI.

## Methods

### Patient selection

The prospective study was approved by the Ethics Committee of the First Affiliated Hospital of Wenzhou Medical University. All study subjects provided informed consent. From January 2013 to July 2019, a total of 2465 patients in the Cardiovascular Department of the First Affiliated Hospital of Wenzhou Medical University were diagnosed with ACS and underwent PCI.

ACS is diagnosed according to current American Heart Association (AHA)/American College of Cardiology (ACC) guidelines, including STEMI and non-ST-segment elevation acute coronary syndrome (NSTE-ACS) [22, 23]. STEMI was defined as continuous chest pain that lasted > 30 min, arrival at the hospital within 12 h from the onset of symptoms, ST-segment elevation > 0.1 mV in ≥ 2 contiguous leads or new left bundle-branch block on the 12-lead electrocardiogram, and elevated cardiac markers (creatine kinase-MB or troponin I) [22]. NSTE-ACS included NSTEMI and UA. NSTEMI was defined as ischemic symptoms in the absence of ST-segment elevation on the electrocardiogram with elevated cardiac markers. UA was defined as having newly developed/accelerating chest symptoms on exertion or rest angina within 2 weeks without biomarker release [23]. The exclusion criteria were as follows: (1) chronic coronary syndrome; (2) tumour history; (3) significant comorbidity, trauma, or surgery; (4) incomplete follow-up data; and (5) death within the first month.

According to these inclusion and exclusion criteria, 1986 patients were included in the study. Patients followed for 4 years were randomly divided into the training (n = 1324) and validation sets (n = 662) based on a computer-generated randomly generated allocation sequence. All methods were carried out in accordance with approved guidelines.

### Clinical outcomes definitions

MACE was defined as the end point of this study and refers to all-cause mortality, clinically driven re-vascularization of target lesions, and new or recurrent myocardial infarction and stroke.

### Collection of demographic, clinical, and follow-up data

All data were extracted from the electronic medical record system. Demographic data included sex and age. The preoperative clinical indicators included left anterior descending branch (LAD) stenosis ≥ 50%, left circumflex artery (LCX) stenosis ≥ 50%, right coronary artery (RCA) stenosis ≥ 50% (according to TIMI criteria [16]), three-vessel disease (LAD, LCX and RCA all with stenosis ≥ 50%), serum lactate level, serum brain natriuretic peptide (BNP) level, estimated glomerular filtration rate (EGFR), serum creatinine level, haemoglobin (HB), serum uric acid level, and left ventricular ejection fraction (LVEF). Blood samples were drawn from peripheral venous blood immediately upon admission and tested at the hospital’s central laboratory. The maximum values of lactate and BNP were taken before coronary angiography. EGFR was calculated using the Chronic Kidney Disease Epidemiology Collaboration (CKD-EPI) equation [24]. LVEF was obtained by echocardiography measured in 2D-biplane at hospital admission (before PCI) [25, 26]. Medical history included hypertension, diabetes, peripheral artery stenosis, atrial fibrillation, previous stroke, and kidney disease. Stenosis of the LAD, LCX and RCA was determined by coronary angiography during hospitalization. Killip class I was defined as the absence of congestive heart failure, class II as the presence of rales and/or jugular venous distention, class III as the presence of pulmonary oedema, and class IV as cardiogenic shock.

Regular medical follow-up data were obtained by telephone and clinic visits. Patients in the training set and the validation set were followed up for four years.

### Statistical analysis

Continuous variables with a normal distribution are expressed as the mean ± standard deviation (${\overline{\text{x}}}$ ± s), and Student’s t-tests were used to compare the differences between the training and validation sets. The nonnormally distributed data are described as the median and 25th and 75th percentiles, and comparisons of the two sets were carried out with the Mann–Whitney U‐test. Categorical variables are expressed as frequencies (proportions), and descriptive comparisons were made using Pearson's χ^2^ test or Fisher’s exact test if one of the expected values in the 2 × 2 table was < 5. Differences in the event rates at different time points after PCI were assessed using the χ^2^ test. The associations of these variables with MACEs were identified using Cox proportional hazards regression models. Forward stepwise selection (likelihood ratio) with the Akaike information criterion (AIC) was used to select variables for the multivariable Cox proportional hazards regression models. The results are reported as hazard ratios (HRs) and 95% confidence intervals (CIs). The identified variables based on the results of multivariate analysis were incorporated to construct the nomogram to predict the risk of MACEs at 6 months, 1 year and 4 years after PCI using statistical software (rms in R, version 3.6.2; http://www.r-project.org). With the input of independent risk factors, the nomogram outputs a risk score for each patient.

The area under the receiver operating characteristic curve (AUC) of the time‐dependent receiver operating characteristic curve (tdROC) varies as a function of time [27]. TdROC was estimated for comparing the discrimination (predictive capability) of the nomogram, CADILLAC risk score and GRACE risk score [28]. The accuracy of calibration was assessed by plotting the nomogram-predicted and observed MACE rates based on the population quartiles of predicted risk. In addition, we analysed the potency of this model to stratify patients at high risk for MACEs.

All data management and statistical analysis were performed using SPSS 20.0, MedCalc 19.0.5 and R 3.6.2. X-tile 3.6.1 was used to obtain cut-off values [29]. All tests were performed 2‐tailed at a significance level of 5%.

### Role of the funding source

This research was funded by the National Natural Science Foundation of China (No. 81873468). The sponsor (ZH) played a role in the research design and review.

## Results

### Baseline characteristics of patients and outcomes

A total of 1986 patients with ACS treated with PCI were included in this study after excluding those with missing data. The training set comprised 1324 patients, with 662 patients in the validation set. The baseline characteristics of the patients in the training and validation sets are shown in Table 1. The baseline characteristics were similar between the two sets, except for sex. The percentage of males in the training set was higher than that of the validation set (81.3% vs. 76.4%, *P* = 0.012).

**Table 1:** Baseline demographics and clinical characteristics of patients in the training set and validation set

Variables	Training set (N = 1324)	Validation set (N = 662)	*P* value
*Discrete variables*			
Sex			0.012
Men (%)	1076 (81.3)	506 (76.4)	
Women (%)	248 (18.7)	156 (23.6)	
Three-vessel coronary artery disease			0.648
Yes (%)	375 (28.3)	194 (29.3)	
No (%)	949 (71.7)	468 (70.7)	
LAD stenosis (≥ 50%)			0.346
Yes (%)	1044 (78.9)	534 (80.7)	
No (%)	280 (21.1)	128 (19.3)	
LCX stenosis (≥ 50%)			0.775
Yes (%)	639 (48.3)	315 (47.6)	
No (%)	685 (51.7)	347 (52.4)	
RCA stenosis (≥ 50%)			0.286
Yes (%)	777 (58.7)	405 (61.2)	
No (%)	547 (41.3)	257 (38.8)	
Hypertension			0.773
Yes (%)	741 (56.0)	375 (56.6)	
No (%)	583 (44.0)	287 (43.4)	
Diabetes			0.564
Yes (%)	293 (22.1)	139 (21.0)	
No (%)	1031 (77.9)	523 (79.0)	
Peripheral artery stenosis			0.818
Yes (%)	290 (21.9)	148 (22.4)	
No (%)	1034 (78.1)	514 (77.6)	
Atrial fibrillation			0.900
Yes (%)	90 (6.8)	46 (6.9)	
No (%)	1234 (93.2)	616 (93.1)	
Previous stroke			0.275
Yes (%)	104 (7.9)	43 (6.5)	
No (%)	1220 (92.1)	619 (93.5)	
Kidney disease			0.695
Yes (%)	55 (4.2)	30 (4.5)	
No (%)	1269 (95.8)	632 (95.5)	
Killip class			0.356
I (%)	989 (74.7)	485 (73.3)	
II (%)	187 (14.1)	91 (13.7)	
III (%)	52 (3.9)	23 (3.5)	
IV (%)	96 (7.3)	63 (9.5)	
TIMI flow grades			0.190
I (%)	91 (6.9)	56 (8.5)	
II (%)	12 (0.9)	11 (1.7)	
III (%)	45 (3.4)	17 (2.6)	
IV (%)	1176 (88.8)	578 (87.3)	
Previous cardiac arrest			0.183
Yes (%)	58 (4.4)	38 (5.7)	
No (%)	1266 (95.6)	624 (94.3)	
*Continuous variables*			
Age, year	64.0 (54.0, 73.0)	64.0 (53.0, 73.0)	0.8513
Lactate, mmol/L	2.80 (2.20, 3.70)	2.80 (2.10, 3.70)	0.687
BNP, pg/mL	277.0 (103.0, 671.5)	270.5 (109.0, 755.0)	0.6686
Uric acid, μmol/L	361.0 (300.0, 438.5)	369.0 (305.0, 447.0)	0.0871
LVEF, %	48.0 (43.0, 55.8)	49.0 (43.0, 55.0)	0.9864
EGFR, mL/min/1.73 m^2^	82.8 (61.0, 100.8)	83.7 (58.8, 100.8)	0.6192
Creatinine, μmol/L	83.0 (71.0, 102.0)	82.0 (70.0, 105.0)	0.4397
Haemoglobin, g/L	133.0 (120.0, 144.0)	132.0 (119.0, 143.0)	0.5253
*MACE rate*			
6-month (%)	42 (3.1)	26 (3.9)	0.3763
1-year (%)	50 (3.8)	27 (4.1)	0.7573
4-year (%)	186 (14.0)	89 (13.4)	0.7132

*BNP* brain natriuretic peptide, *EGFR* estimated glomerular filtration rate, *LAD* left anterior descending branch, *LCX* left circumflex artery, *LVEF* left ventricular ejection fraction, *MACE* major adverse cardiovascular events, *RCA* right coronary artery

During follow-up, MACE occurred in 201 (15.1%) cases in the training data set but not in 1123 cases. For the training set, after 6 months, 1 year and 4 years, the MACE rates were 3.1%, 3.8% and 14.0%, respectively. For the validation set, MACE occurred in 96 (14.5%) cases but not in 566. The MACE rates in the validation set 6 months, 1 year and 4 years later were 3.9%, 4.1% and 13.4%, respectively. The χ^2^ test showed no significant differences between the two groups (*P* > 0.05) (Table 1).

### Development of the multivariate prognostic nomogram

**Fig. 1:**
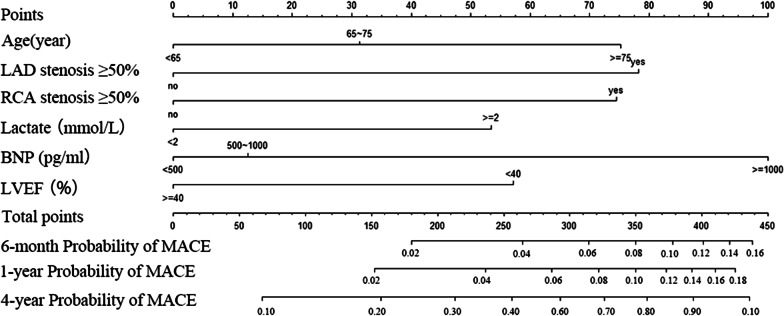
Nomogram for predicting MACEs in patients with ACS after PCI. Points were assigned for age, LAD stenosis ≥ 50%, RCA stenosis ≥ 50%, lactate, BNP, and LVEF. The score for each value was assigned by drawing a line upward to the points line, and the sum of the six scores was plotted on the total points line. Finally, the probability line was used to determine the probability of MACE

According to the univariate Cox regression analysis, 15 candidate clinical variables were found to meet the threshold of *P* < 0.05 (Table 2). The multivariate Cox regression analysis indicated that age, LAD stenosis, RCA stenosis, lactate, BNP and LVEF were significant independent predictors of the MACE rate in the training set (*P* < 0.05). These predictors were used to construct the prediction model (Fig. 1). Each predictor corresponded to a specific point by drawing the straight line upwards to the point axis. Scores for each variable were summed and located on the “Total Points” axis. Finally, a vertical line was drawn straight down from the plotted total point axis to the probability axis to locate the likelihood of MACE.

**Table 2:** Univariate and multivariable Cox hazards analysis of the training cohort

Variables	Univariate		Multivariate		Score
	HR(95% CI)	*P* value	HR(95% CI)	*P* value	
*Statistically significant factors*					
Age, year					
< 65	Ref	Ref	Ref	Ref	0
65–75	1.636 (1.157–2.314)	0.005	1.295 (0.909–1.847)	0.153	31
≥ 75	2.807 (2.015–3.910)	< 0.001	1.866 (1.307–2.663)	0.001	75
LAD stenosis ≥ 50%	2.192 (1.445, 3.327)	< 0.001	1.909 (1.247–2.925)	0.003	78
RCA stenosis ≥ 50%	1.969 (1.444, 2.684)	< 0.001	1.854 (1.350–2.545)	< 0.001	74
Lactate ≥ 2 mmol/L	1.604 (1.096, 2.347)	0.015	1.555 (1.051–2.299)	0.027	53
BNP, pg/mL					
< 500	Ref	Ref	Ref	Ref	0
500–1000	1.467 (1.002, 2.150)	0.049	1.105 (0.744–1.642)	0.621	12
≥ 1000	3.506 (2.567, 4.789)	< 0.001	2.284 (1.620–3.219)	< 0.001	100
LVEF < 40%	2.138 (1.585, 2.885)	< 0.001	1.607 (1.167–2.211)	0.004	57
Men	0.508 (0.375, 0.689)	< 0.001			
LCX stenosis ≥ 50%	1.394 (1.051, 1.848)	0.021			
Hypertension	1.523 (1.137, 2.040)	0.005			
Diabetes	1.310 (0.952, 1.803)	0.097			
Atrial fibrillation	1.841 (1.223, 2.771)	0.003			
Kidney disease	2.284 (1.367, 3.814)	0.002			
EGFR < 60 mL/min/1.73 m^2^	2.149 (1.618, 2.855)	< 0.001			
Creatinine > 186 μmol/L	2.402 (1.566,3.684)	< 0.001			
Haemoglobin < 120 g/L	1.831 (1.370, 2.448)	< 0.001			
*Statistically non-significant factors*					
Peripheral artery stenosis	1.290 (0.928, 1.793)	0.129			
Previous stroke	1.419 (0.924, 2.179)	0.109			
Previous cardiac arrest	1.193 (0.611, 2.331)	0.605			
Uric acid, μmol/L	1.310 (0.926, 1.854)	0.128			

*BNP* brain natriuretic peptide, *EGFR* estimated glomerular filtration rate, *LAD* left anterior descending artery, *LCX* left circumflex artery, *LVEF* left ventricular ejection fraction, *RCA* right coronary artery

### Assessment of the nomogram’s performance

**Fig. 2:**
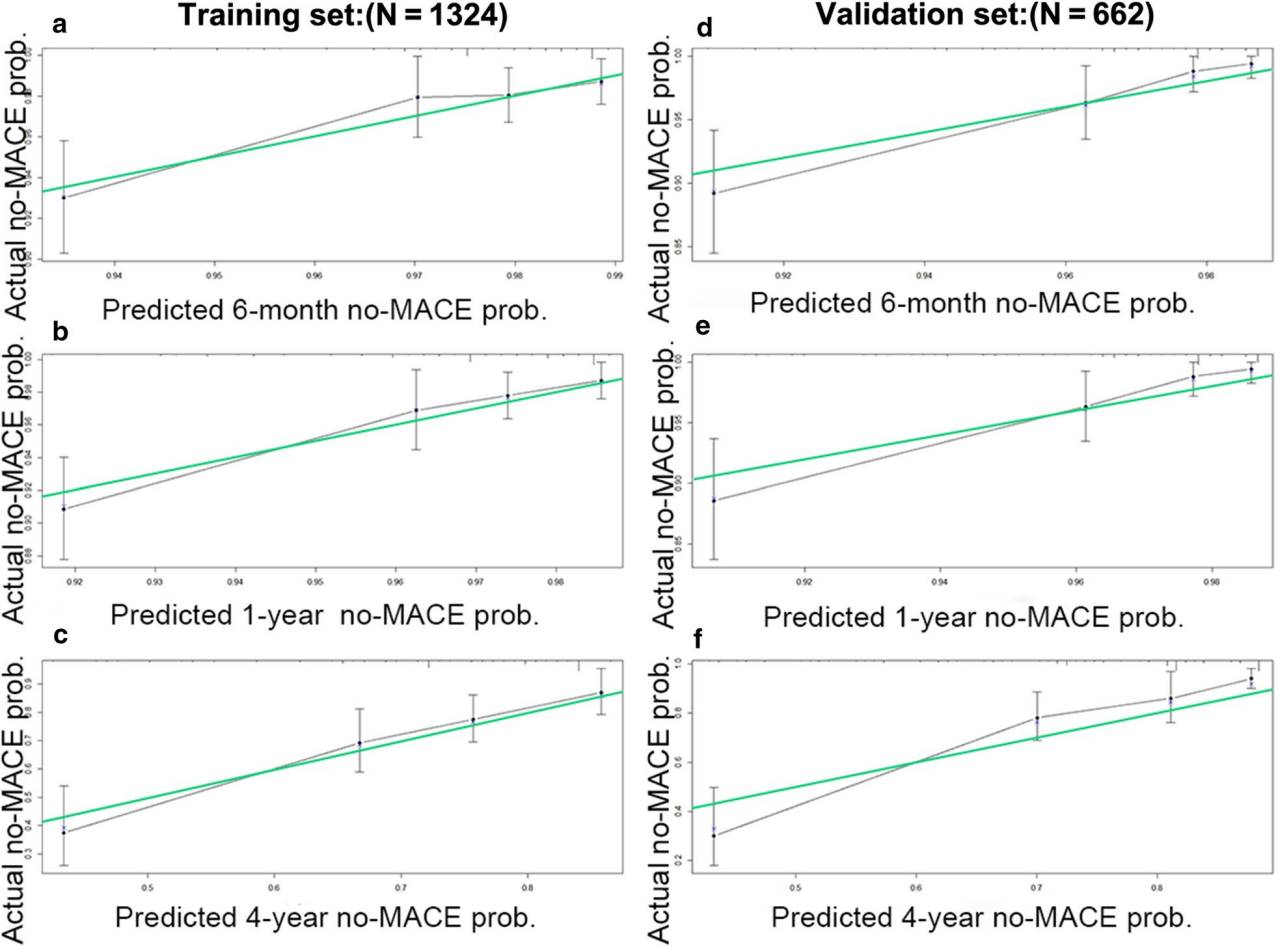
The calibration curve for predicting MACE probability. **a** 6 months, **b** 1 year, and **c** 4 years in the training set; **d** 6 months, **e** 1 year, and **f** 4 years in the validation set. The nomogram-predicted probability of no MACE is plotted on the X-axis; the actual probability is plotted on the Y-axis

In the training set, the 6-month area under the ROC curve (AUC) was 0.712 (95% CI 0.621–0.803) for the model, the 1-year AUC was 0.741 (95% CI 0.665–0.817), and the 4-year AUC was 0.762 (95% CI 0.692–0.831), indicating excellent discrimination (Table 2). The calibration of predictions from the model was satisfactory, as assessed by comparison of prediction by nomogram to the actual MACE rate across quartiles of risk, as shown in Fig. 2a–c.

### Validation of the nomogram

In the validation set, the AUC at 6 months was 0.811 (95% CI 0.730–0.891), the 1-year AUC was 0.818 (95% CI 0.739, 0.897), and the 4-year AUC was 0.724 (95% CI 0.631–0.816) (Table 3). The favourable calibration of the nomogram was also confirmed in the validation set (Fig. 2d–f).

**Table 3:** Comparisons of AUCs of the risk scores to predict MACEs

Time	Risk scores	Training set			Validation set		
		AUC	95% CI	*P* value	AUC	95% CI	*P* value
6 months	Nomogram	0.712	0.621–0.803	Ref	0.811	0.730–0.891	Ref
	CADILLAC score	0.674	0.582–0.766	0.2840	0.715	0.605–0.825	0.0044
	GRACE score	0.653	0.556–0.751	0.1519	0.75	0.659–0.842	0.0351
1 year	Nomogram	0.741	0.665–0.817	Ref	0.818	0.739–0.897	Ref
	CADILLAC score	0.699	0.622–0.775	0.1670	0.725	0.617–0.833	0.0043
	GRACE score	0.662	0.578–0.746	0.0360	0.761	0.672–0.850	0.0390
4 years	Nomogram	0.762	0.692–0.831	Ref	0.724	0.631–0.816	Ref
	CADILLAC score	0.572	0.496–0.648	< 0.0001	0.629	0.534–0.724	0.0024
	GRACE score	0.629	0.549–0.710	0.0003	0.622	0.522–0.722	0.0209

*AUC* area under the curve, *CADILLAC* controlled abciximab and device investigation to lower late angioplasty complications, *CI* confidence interval, *GRACE* global registry of acute coronary events, *MACE* major adverse cardiovascular events

### Comparing the performance of the newly developed risk score with existing risk scores

**Fig. 3:**
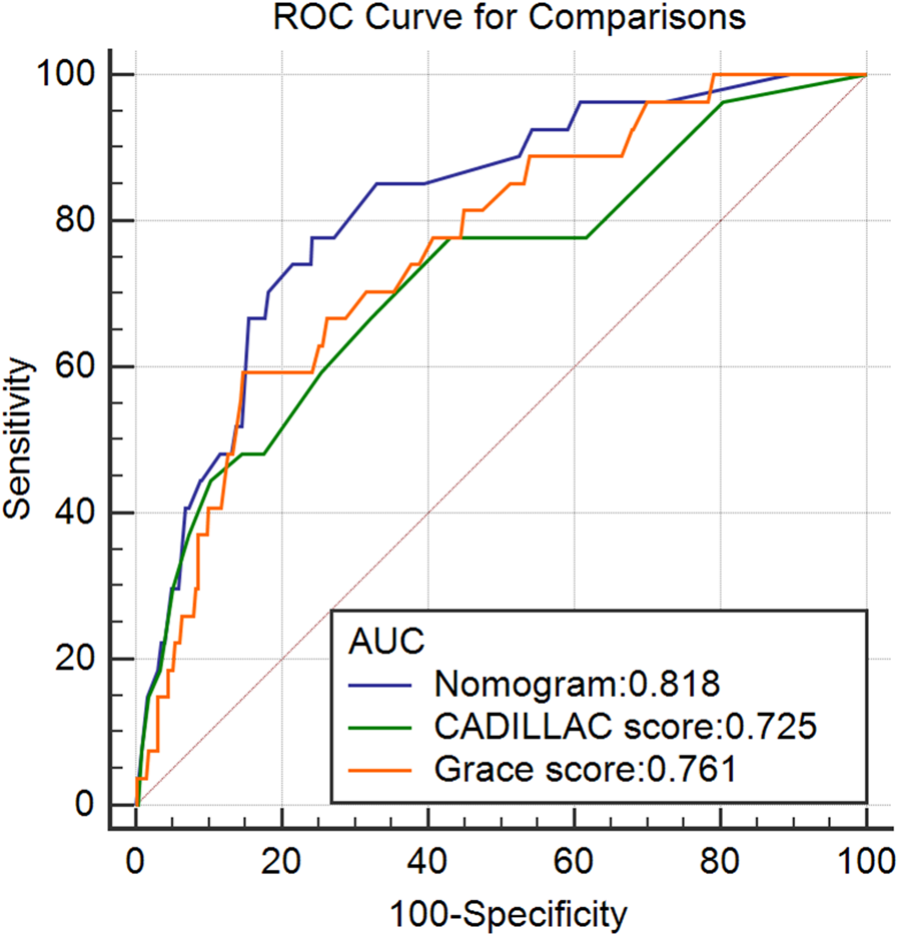
Time-dependent ROC curve (tdROC) for the nomogram, CADILLAC score and GRACE score. Performance comparison was assessed between the nomogram, CADILLAC score and GRACE score by calculating the area under the ROC curves in the validation set for 1-year MACE. *CADILLAC* the Controlled Abciximab and Device Investigation to Lower Late Angioplasty Complications study, *GRACE* the Global Registry of Acute Coronary Events, *ROC* receiver operating characteristics

In the training and validation sets, we compared the tdROCs of the nomogram with the CADILLAC score and GRACE score. The results showed that the discrimination of the nomogram was most favourable (Table 3; Fig. 3).

### Performance of the prognostic nomogram in stratifying risk

**Fig. 4:**
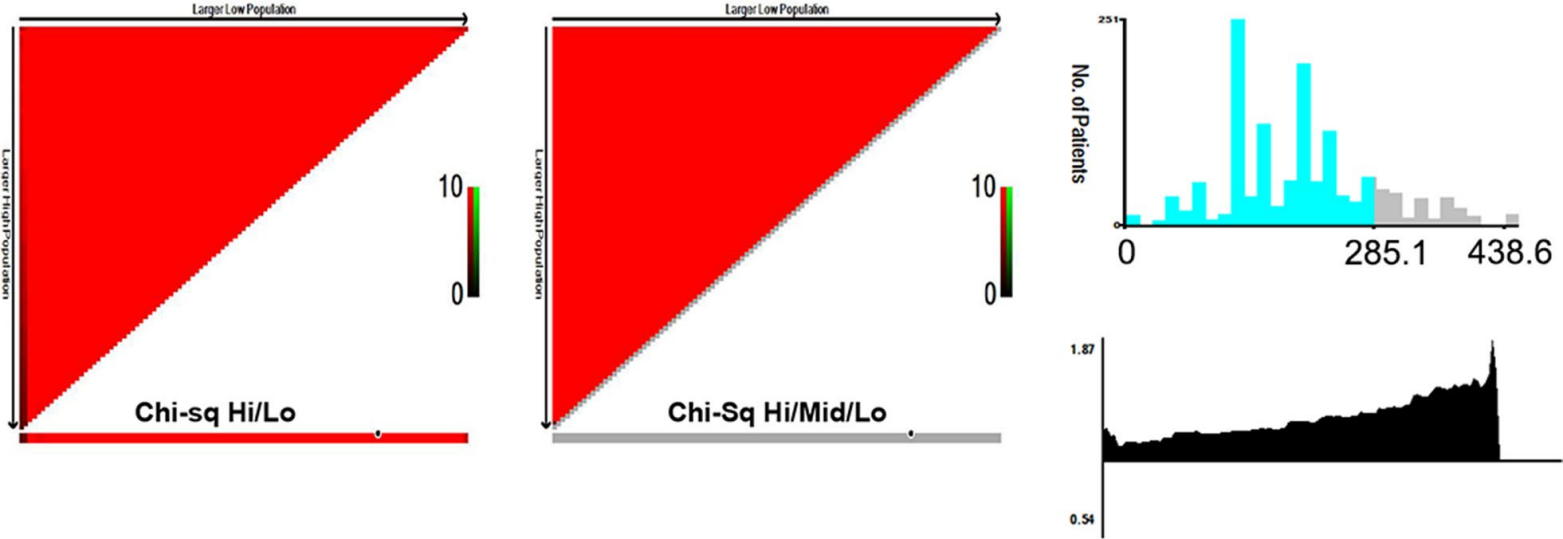
X-tile analysis of the total risk score in the training set and cut-off value. The optimal cut-off value for the total risk score was 285.1 (χ^2^ = 99.0394, *P* < 0.001)

In the training set with an endpoint time of 4 years, the total prognostic scores calculated by the nomogram were categorized into two risk groups to predict MACE: ‘low-risk’ (score ≤ 285.1) and ‘high-risk’ (score > 285.1) based on the cut-off value calculated using X-tile software [28] (Fig. 4).

**Fig. 5:**
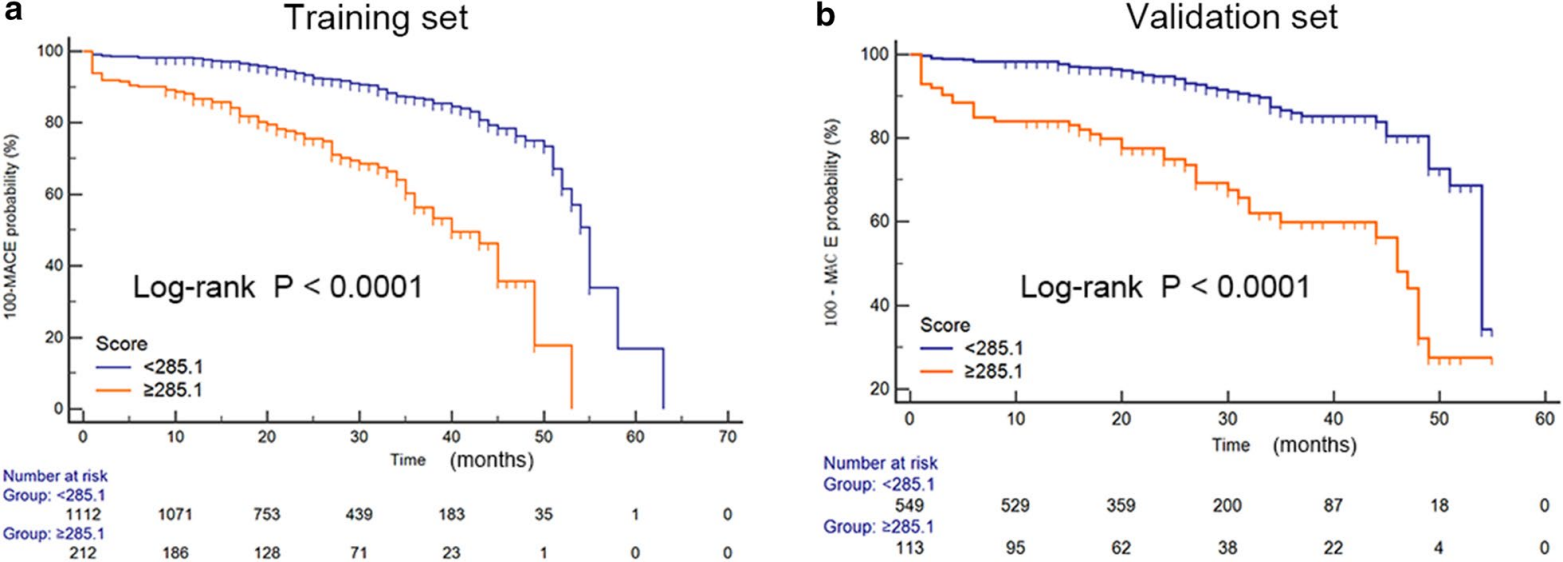
Kaplan–Meier survival curves in the training (**a**) and validation sets (**b**), stratified by the nomogram (‘high-risk’ [score ≥ 285.1] and ‘low-risk’ [score < 285.1))

The Kaplan–Meier curves for both sets clearly showed that the nomogram was stable in differentiating between high-risk and low-risk patients (Fig. 5). The HR for the ‘high-risk’ category was found to be 4.11 (95% CI 3.08–5.49) compared to the ‘low-risk’ category in the training set and 4.01 (95% CI 2.68–6.00) in the validation set.

## Discussion

Our results confirmed that several demographic and clinical characteristics reported from previous models developed from other databases can be used to construct a simple model for prognostic evaluation. The 6 most important factors—lactate level, age, LAD stenosis, RCA stenosis, BNP level, and LVEF—contained most of the prognostic information and were incorporated into the nomogram. To our knowledge, this nomogram is the first clinical prediction model incorporating lactate for predicting the long-term risk of MACEs among patients with ACS after PCI. Nomograms are evidence-based and fully personalized tools to guide clinical decision-making and provide patient-friendly, accurate and repeatable predictions without the need for computer software to interpret [30]. Risk stratification is important in determining which medications and revascularization should be used. In addition, the prediction contributes to the development of health care and clinical guidelines for ACS.

Lactate, as an easily and quickly assessed metabolite, has been studied over time in acute cardiac patients to evaluate its prognostic ability [19]. A meta-analysis showed a greater reduction in lactate concentrations in survivors than in non-survivors, whether following cardiac surgery, cardiogenic shock, or cardiac arrest [31]. Harjola et al. found that lactate level (> 2 mmol/L) was independently associated with increased short-term mortality for patients with cardiogenic shock [32]. For patients with STEMI, higher lactate levels were independently associated with 30-day mortality and overall adverse reactions to PCI (in particular, lactate ≥ 1.8 mmol/L) [33]. Marashly et al. found that for patients with cardiogenic shock secondary to acute coronary syndrome, lactate ≥ 2.5 mmol/L could independently predict 30-day all-cause mortality and then established an ACS-MCS score [34]. Ruling out cardiogenic shock, in 766 patients with STEMI and NSTEMI undergoing coronary artery bypass surgery, lactate was a predictor of 30-day and late mortality [35]. In addition, in a study of 1865 patients with ACS, elevated lactate levels (≥ 1.8 mmol/L) at admission were an independent predictor of 30-day and 180-day all-cause mortality [18]. Lactate is an important fuel for the stressed heart and is produced by the dehydrogenation of pyruvate, which is synthesized from glycolysis [36, 37]. In a normal heart at rest, β-oxidation of fatty acids provides approximately 60–90% of energy, while pyruvate produces 10–40% [38]. During exercise, the uptake and use of lactate in the myocardium increases, as does the stimulation of β-adrenergic stimulation and shock [39]. Hyperlactatemia can be seen as part of the stress response, including increased metabolic rate, sympathetic nervous system activation, accelerated glycolysis, and improved bioenergy supply [19]. Hyperlactate after ACS may be caused by hypoxia following haemodynamic disorders or by catecholamine-induced aerobic glycolysis in response to stress [39, 40]. These studies suggest that lactate may play an important role in the course of ACS. To the best of our knowledge, however, there has been no risk prediction tool for MACEs integrating lactate to date. Therefore, a well-performed risk prediction model incorporating lactate is urgently needed. It must be acknowledged that certain drugs (e.g., metformin, sodium bicarbonate), preoperatively sustained fasting periods with volume depletion, and even hypertension could have contributed to changes in lactate levels, which could interfere with this evaluation [35]. Unfortunately, we did not calculate lactate clearance, which has been reported to be more reliable on clinical grounds than lactate for risk stratification in different critical illness conditions [41–43].

For the other five variables, TIMI risk score indicated prior coronary stenosis of 50% or more as an independent predictor for the primary end point (all-cause mortality, MI, or urgent revascularization) occurred by 14 days [16]. In a study of 6755 patients after PCI, Iqbal et al. found that for patients with multivessel disease, untreated proximal LAD and RCA (stenosis > 70%) w ere associated with increased mortality [44]. BNP level was a strong independent predictor of short-term postoperative mortality [45]. Grabowski et al. improved their model’s predictive power by adding BNP to the Killip class and TIMI flow grades [46]. A possible explanation is that an elevated BNP level reflects a larger infarct size and progressive left ventricular remodelling, thus more obviously reflecting the degree of cardiac insufficiency [47]. Similar to BNP, LVEF also serves as a reference index for cardiac function to supply important prognostic information and should be included in approaches for stratifying risk after myocardial infarction [48, 49] Many studies have reported that age is a significant risk factor for clinical events (cardiac death, target vessel myocardial infarction, and clinically driven target vessel revascularization) after PCI [50, 51]. The predictive ability of simple age cut-off points of 65 and 75 are similar to that of a more complex model with age as a continuous variable [16]. To overcome or avoid the limitations of a single predictor and achieve high prediction accuracy, we combined six detected predictors into this model. Because of dynamic variations, the nomogram did not include clinical symptoms and signs, such as Killip class, heart rate, and systolic blood pressure, which are significantly associated with ACS mortality [7, 8, 14, 16]; Killip class may result in information bias by the judgement error of the clinician’s supervisor. Nomograms are easy to recall and clinically useful.

The model had adequate discrimination and calibration power in the training set (AUC = 0.712–0.762) to predict MACEs and appeared statistically robust in that it was validated in a separate third of the participants (AUC = 0.724–0.818). Discrimination for MACE prediction of the nomogram was superior to GRACE risk score and CADILLAC risk score in both sets, confirming that nomogram was more valuable in predicting MACEs, especially in the long term. The TIMI risk score, published in 2000, predicted the primary end point (all-cause mortality, MI, or severe recurrent ischaemia requiring urgent revascularization) through 14 days after randomization for UA/NSTEMI [52]. The GRACE risk score has been established to predict the risk of death during hospitalization and at 6 months for patients with ACS [7]. To predict 30-day and 1-year mortality risk after PCI for AMI, the PAMI risk score and CADILLAC risk score were established successively [8, 14]. Several studies have proven that in predicting 30-day and 1-year mortality, the CADILLAC risk score showed slight superiority over GRACE, TIMI, and PAMI risk scores [53–55]. The probable reason is that the CADILLAC risk score emphasizes the importance of LVEF and three-vessel disease [54]. Our nomogram also incorporated these variables. Their predictors, such as heart rate, systolic blood pressure, myocardial enzymes and creatinine, are dynamic. Killip class, postprocedural TIMI flow grade, and ST-segment deviation require the judgement of professional physicians. In addition, previous risk models were derived from Western populations, which limited their application to other populations. In addition, few of the participants were followed up for more than one year.

In regard to the clinical application of the nomogram, we have taken an example of a case of 75 years old (75 points), LAD stenosis 50% (78 points), RCA stenosis 10% (0 point), lactate 1 mmol/L (0 point), BNP 100 pg/ml (0 point) and LVEF 38% (57 points). The total score was 210, and the expected MACE rates after 6 months, 1 year and 4 years were 2%, 3% and 30%, respectively. Patients with ACS undergoing PCI can be thus classified into high- and low-risk groups for 4-year MACEs.

The most attractive aspect features of these models are their accuracy, generalizability, and ease of use. The nomogram is an excellent model to span the entire spectrum of ACS. It is based on a relatively unselected group of patients, representing patients seen in general clinical practice. It includes a new variable, lactate level, that is stable and easily accessible. In addition, the nomogram has an excellent ability to discriminate risk. In the past, risk scores were mostly based on Western populations, while the population of patients with ACS after PCI in the East, especially in China, was much larger, requiring a specialized prediction model. The nomogram uses the latest clinical data from the past 7 years to reflect the current cardiovascular medical level. Although ACS can be treated in many ways, our study evaluated patient outcomes solely treated with PCI, with fewer uncontrolled variables and more stable clinical events. Unlike traditional risk scores, a follow-up period of up to 4 years is conducive to the evaluation of long-term prognosis.

Limitations also existed in this study. Although lactate has certain predictive ability, the detection time and collection method of lactate are not unified and clear. Some clinical drugs may cause changes in lactate without improving the prognosis. Besides, the role of lactate may not be consistent in a general cohort of ACS patients including STEMI, NSTEMI and UA. Since it clearly indicates those patients with hemodinamic compromise and more probably STEMI patients. Subgroup analyses of STEMI, NSTEMI and UA were not performed for lack of adequate detailed information of all patients, resulting in the prediction performance of the model in this three cohorts not being estimated separately. A multi-center validation study, particularly involving other ethnic groups, is required to confirm the performance of the nomogram before clinical application.

## Conclusions

In conclusion, a novel prognostic nomogram incorporating lactate level and five other easily available and objective variables can serve as an accurate and favourable prognostic prediction of 6-month, 1-year, and 4-year incidence of MACEs among patients with the entire spectrum of ACS after PCI. This information can help clinicians stratify risk for optimal triage and management.

## Data Availability

The datasets used and/or analysed during the current study are available from the corresponding author upon reasonable request.

## Abbreviations

ACS: Acute coronary syndrome
AMI: Acute myocardial infarction
AUC: Area under the receiver operating characteristic curve
BNP: Brain natriuretic peptide
CI: Confidence interval
CKD-EPI: Chronic Kidney Disease Epidemiology Collaboration
EGFR: Estimated glomerular filtration rate
HB: Haemoglobin
HR: Hazard ratio
LAD: Left anterior descending branch
LCX: Left circumflex artery
LVEF: Left ventricular ejection fraction
MACE: Major adverse cardiovascular events
NSTE-ACS: Non-ST-segment elevation acute coronary syndrome
NSTEMI: Non-ST-segment elevation myocardial infarction
PCI: Percutaneous coronary intervention
RCA: Right coronary artery
ROC: Receiver operating characteristic
STEMI: ST-segment elevation myocardial infarction
tdROC: Time‐dependent receiver operating characteristic curve
UA: Unstable angina
